# Ultrasound aspects of symptomatic versus asymptomatic forms of male accessory gland inflammation

**DOI:** 10.1111/andr.13014

**Published:** 2021-05-06

**Authors:** Sandro La Vignera, Andrea Crafa, Rosita A. Condorelli, Federica Barbagallo, Laura M. Mongioì, Rossella Cannarella, Michele Compagnone, Antonio Aversa, Aldo E. Calogero

**Affiliations:** ^1^ Department of Clinical and Experimental Medicine University of Catania Catania Italy; ^2^ Department of Experimental and Clinical Medicine Magna Graecia University of Catanzaro Catanzaro Italy

**Keywords:** ejaculatory disorders, erectile dysfunction, MAGI, questionnaire, symptoms, ultrasound

## Abstract

**Background:**

The ultrasound (US) evaluation of the male sex accessory gland inflammation (MAGI) helps the clinicians to understand the severity of this condition, allowing them to distinguish the uncomplicated form (prostatitis) from the complicated ones (prostate‐vesiculitis and prostate‐vesiculitis‐epididymitis), as well as the unilateral from the bilateral forms, the fibrosclerotic and the hypertrophic‐congestive form.

**Objective:**

This retrospective study aimed to evaluate the US features of MAGI patients with active symptoms compared to patients without sexual, voiding, and ejaculatory dysfunction.

**Materials/Methods:**

To achieve this aim, an analysis of the prevalence of MAGI US criteria was carried out on a very large series of over 500 patients diagnosed with MAGI classified according to the different symptom profile evaluated through a dedicated questionnaire (previously conceived and published by our group) arbitrarily named “structured interview about MAGI” (SI‐MAGI) for sexual, voiding, and ejaculatory disorders reported by these patients.

**Results:**

The results of this study revealed that US criteria most frequently detected in patients with severe urinary symptoms were the presence of areas of high echogenicity (almost exclusively in the periurethral prostatic zone) together with the presence of single or multiple areas of acinar ectasia of the prostate. The presence of seminal vesicles with polycyclic areas within the glandular lumen separated by hyperechoic septa represented US criterion most frequently detected in patients with severe spontaneous or post‐ejaculate pain. Finally, US criterion most frequently detected in patients with severe sexual dysfunction was the dilation of the periprostatic venous plexus, suggesting the hypothesis of a possible alternative therapeutic approach.

**Conclusion:**

The data of the present study suggest that symptoms may associate with US signs in patients with MAGI. Also, specific US signs may associate with specific symptoms. Further studies are needed to understand whether patients with specific US signs may in turn benefit from a personalized therapeutic choice.

## 1 INTRODUCTION

Male accessory gland inflammation (MAGI) represents an acronym applied in clinical practice to describe the inflammations of the male accessory sex glands. In detail, prostate, epididymis, and seminal vesicles represent the anatomical sites potentially involved. MAGI, which usually has a chronic course, is able to negatively impact on the semen quality and can associate with a wide range of symptoms.^1^, ^2^, ^3^, ^4^, ^5^

Diagnosis of MAGI is based on widely accepted clinical and laboratory criteria.^2^, ^3^, ^5^, ^6^ However, ultrasound (US) examination can provide details regarding the anatomical extension of the inflammatory process.^1^, ^7^, ^8^, ^9^

The symptoms of patients with MAGI are not easily assessable and, notably, patients are falsely asymptomatic in several cases. The true reasons for the first clinical counseling could concern different issues, such as ejaculatory or voiding function, quality of sexual life, and chronic pelvic pain.^10^ For this reason, we have previously adopted a specific questionnaire, arbitrarily named “structured interview about MAGI” (SI‐MAGI), which includes different symptom domains and which could guide the clinician through the collection of the patients’ medical history.^10^

The first level of clinical evaluation should include: 1. an active collection of medical history (supplemented by questionnaire); 2. physical andrological examination (with careful evaluation of the prostatic region through anorectal digital exploration); 3. semen analysis; 4. microbiological evaluation of secretion obtained after prostate massage.^1^, ^3^ The incomplete application of this procedure involves a diagnostic underestimation of the diagnosis of MAGI and explains the reasons for the different frequency reported in the various clinical studies.^11^, ^12^, ^13^ In our experience, together with varicocele, MAGI represents the condition with the greatest prevalence in infertile patients. The incompleteness of the clinical evaluation of patients with MAGI may be likely ascribed to the lack of urologic and endocrinologic expertise. Indeed, these kinds of patients usually refer to endocrinologists or urologists. While endocrinologists may have difficulty with anorectal digital exploration, urologists may have an incomplete seminological training being more trained in surgical practice.^1^ Indeed, endocrinologists with urologic expertise or, in contrast, urologists with endocrinologic competence, are lacking.

In clinical practice, the application of US criteria for the study of infertile patients is generally limited to the exclusion of obstructive azoospermia.^14^ In our experience, the targeted use of US evaluation of patients with MAGI allows us to make a diagnosis of the anatomical site, helping in the distinction of unilateral or bilateral forms, or in the description of forms with better or worse seminological prognosis. It can also help in the differential diagnosis of some highly associated sexual dysfunctions, such as acquired premature ejaculation or the evaluation of cases of hemospermia.^1^

Based on the premises, the present study evaluated the US differences between symptomatic and asymptomatic patients with diagnosis of MAGI (according to the questionnaire scores). To accomplish this, the US features of patients with MAGI were retrospectively reviewed, and the findings were analyzed according to the SI‐MAGI questionnaire score.

## 2 MATERIALS AND METHODS

The outpatient medical records of 1320 patients with a diagnosis of MAGI were retrospectively evaluated. Patients referred to the Andrology and Endocrinology Center for infertility, varicocele, fertility checking, phimosis, urogenital infection or inflammation, and scrotal pain.

The diagnosis of MAGI was established using the criteria of the World Health Organization. Particularly, patients were diagnosed for MAGI in case of oligo‐, astheno‐, and/or teratozoospermia associated with one factor A plus one factor B, or one factor A plus one factor C, or one factor B plus one factor C, or two factors C. Factor A occurred in case of history positive for urinary infection, epididymitis, and/or sexually transmitted diseases, or in the presence of physical signs of urogenital inflammation (eg, thickened or tender epididymis, tender vas deferens, and/or abnormal digital rectal examination). Factor B occurred in case of abnormal prostate fluid expression and/or abnormal urine after prostatic massage. Factor C occurred in case of ejaculatory signs of inflammation (leukocyte > 1 million/ml, culture with significant growth of pathogenic bacteria, abnormal appearance, increased viscosity, increased pH, and/or abnormal biochemistry of the seminal plasma).^1^, ^2^, ^3^ Patients were enrolled in the period between January 2011 and January 2020 at the Andrology and Endocrinology Center of the University of Catania. The US of the male accessory glands was performed by specialists specifically trained in the identification of suggestive US feature of MAGI (SLV, AEC, AC, MC).

All patients for whom access to the US report was not available or who had not compiled or completed the SI‐MAGI questionnaire were excluded from the analysis. Figure S1 summarizes the questions of the questionnaire administered to patients.

Patients were grouped basing on the specific symptom they complained of. Specifically, the US characteristics of 525 patients with MAGI aged between 20 and 43 years were analyzed, and patients were divided into:

- *Group A (no. 170)*: MAGI associated with severe urinary symptoms (domain questionnaire no. 1 score > 13).
- *Group B (no. 190)*: MAGI associated with severe spontaneous or post‐ejaculatory pain/discomfort (domain questionnaire no. 2 score > 17).
- *Group C (no. 165)*: MAGI associated with severe sexual dysfunction (domain questionnaire no. 3 score > 23).

Patients complaining of combined symptoms (eg, severe urinary symptoms plus severe spontaneous post‐ejaculatory pain or severe sexual dysfunction) were excluded from the analysis.

For each group, the observational frequency of the various US criteria adopted for the diagnosis of MAGI was calculated (no. 6 US criteria suggestive for chronic prostatitis; no. 8 US criteria suggestive for chronic vesiculitis; no. 6 US criteria suggestive for chronic epididymitis).^1^, ^7^

Within Group C, a further distinction was made regarding the observational frequency of the aforementioned US criteria detected in men with erectile dysfunction in the achievement and maintenance phase and in patients with ejaculatory disorders.

A series of 120 patients with MAGI between 18 and 48 years without symptoms (questionnaire scores lower than the minimum in all domains of the questionnaire) were considered as the control group.

### 2.1 Statistical analysis

Results are shown as percentage. Statistical analysis was performed by one‐way analysis of variance (ANOVA), followed by the Duncan's multiple range test, using SPSS 22.0 for Windows (22.0, SPSS Inc). A *p* value < 0.05 was accepted as statistically significant.

### 2.2 Ethical approval

This study was conducted at the Division of Andrology and Endocrinology of the teaching hospital “G. Rodolico”, University of Catania (Catania, Italy). informed written consent was obtained from each participant after full explanation of the purpose and nature of all procedures used. The study has been conducted in accordance with the principles expressed in the Declaration of Helsinki.

## 3 RESULTS

US criteria most frequently detected in patients with severe urinary symptoms (Group A) were the presence of areas of high echogenicity (also defined as calcifications in clinical practice) together with the presence of single or multiple areas of acinar ectasia of the prostate (box highlighted in yellow in Table 1. The combination of these two US criteria was found in 58% of cases. Among 100 patients with this US combination, 90% (90) of them showed a periurethral localization of the calcifications.

**TABLE 1:** Frequencies of US criteria suggestive for MAGI reported in the three examined groups

US parameter suggestive for chronic prostatitis (P1–P6), for chronic vesiculitis (V1–V8), for chronic epididymitis (E1–E6)	Group A	Group B	Group C	Controls
Asymmetry of the gland volume (P1)	40/170 (23.5%)^^^	20/190 (10.5%)	20/165 (12.1%)	6/120 (5.0%)
Areas of low echogenicity (P2)	20/170 (11.8%)	40/190 (21.0%)	30/165 (18.1%)	10/120 (8.3%)
Areas of high echogenicity (P3)	100/170 (58.8%)^*^	80/190 (42.1%)^^^	65/165 (39.3%)	10/120 (8.3%)
Dilatation of periprostatic venous plexus (P4)	40/170 (23.5%)	25/190 (13.1%)	100/165 (60.6%)^*^	8/120 (6.6%)
Single or multiple internal similar cystic areas (P5)	100/170 (58.8%)*	90/190 (47.3%)^^^	40/165 (24.2%)	12/120 (10.0%)
Area/s of moderate increase of vascularity (P6)	22/170 (12.9%)	20/190 (10.5%)	20/165 (12.1%)	6/120 (5.0%)
Increase anteroposterior diameter (mono‐ or bilateral) (V1)	22/170 (12.9%)	25/190 (13.1%)	30/165 (18.1%)	8/120 (6.6%)
Asymmetry compared to the controlateral seminal vesicle (V2)	20/170 (11.8%)	25/190 (13.1%)	20/165 (12.1%)	8/120 (6.6%)
Reduced anteroposterior diameter (mono‐ or bilateral) (V3)	40/170 (23.5%)^^^	40/190 (21.0%)	18/165 (10.9%)	3/120 (2.5%)
Glandular epithelium thickened and/or calcified (V4)	20/170 (11.8%)	30/190 (15.7%)	20/165 (12.1%)	8/120 (6.6%)
Polycyclic areas separated by hyperechoic septa in one or both Vesicles (V5)	60/170 (35.2%)	120/190 (63.1%)^*^	45/165 (27.3%)	6/120 (5.0%)
Fundus/body ratio > 2.5 (V6)	50/170 (29.4%)	60/190 (31.6%)^^^	40/165 (24.2%)	6/120 (5.0%)
Fundus/body ratio < 1 (V7)	22/170 (12.9%)	40/190 (21.0%)^^^	18/165 (10.9%)	3/120 (2.5%)
Anteroposterior diameter unchanged after ejaculation (V8)	30/170 (17.6%)	45/190 (23.7%)	75/165 (45.4%)	10/120 (8.3%)
Increase in size of the head and/or of the tail (single or bilateral) (E1)	60/170 (35.2%)	90/190 (47.3%)^^^	20/165 (12.1%)	6/120 (5.0%)
Presence of multiple microcystis in the head and/or tail (finding single or bilateral) (E2)	20/170 (11.8%)	50/190 (26.3%)	30/165 (18.1%)	8/120 (6.6%)
Low echogenicity or high echogenicity mono‐ or bilateral (E3)	30/170 (17.6%)	60/190 (31.6%)	40/165 (39.3%)^^^	6/120 (5.0%)
Large hydrocele mono‐ or bilateral (E4)	30/170 (17.6%)	30/190 (15.8%)	40/165 (24.2%)^^^	3/120 (2.5%)
Enlargement in superior part of the cephalic tract and superior/inferior part ratio > 1 (E5)	50/170 (29.4%)^^^	20/190 (10.5%)	25/165 (15.2%)	5/120 (4.2%)
Unchanged anteroposterior diameter of tail after ejaculation (E6)	25/170 (14.7%)	30/190 (15.8%)	75/165 (45.4%)^^^	12/120 (10.0%)

^*^ *p* < 0.01 versus other US criteria examined within the same group (yellow panels).

^ *p* < 0.05 versus controls (green panels).

Compared to patients with severe spontaneous or post‐ejaculatory pain or severe sexual dysfunction, an higher, but not statistically significant, frequency of the following US criteria was found: glandular asymmetry and presence of areas with increased vascularization of the prostate, reduction of interparietal thickness of the seminal vesicles, and increased transverse diameter of the upper cephalic tract of the epididymis.

US criterion most frequently detected in patients with severe spontaneous or post‐ejaculate pain (Group B) was the presence of seminal vesicles with polycyclic areas within the glandular lumen separated by hyperechoic septa (63% of cases) (box highlighted in yellow in Table 1. Compared to patients with severe urinary symptoms and severe sexual dysfunction, a higher frequency, but not statistically significant, of the following US criteria was found: areas of reduced echogenicity of the prostate, asymmetry between the two seminal vesicles, thickening and/or presence of calcifications of the seminal vesicles glandular epithelium, alteration of the body/fundus ratio of the seminal vesicles, increase in size of the cephalic tract or tail of the epididymis, and presence of epididymal microcysts.

US criterion most frequently detected in patients with severe sexual dysfunction (Group C) was the dilation of the periprostatic venous plexus (60% of cases) (box highlighted in yellow in Table 1. Compared to patients with severe urinary symptoms and severe spontaneous or post‐ejaculatory pain, a higher frequency, but not statistically significant, of the following US criteria was found: dilation of seminal vesicles maintained after ejaculation,changes in echogenicity of the epididymis; presence of hydrocele; epididymal dimensions unchanged after ejaculation.

**FIGURE 1:**
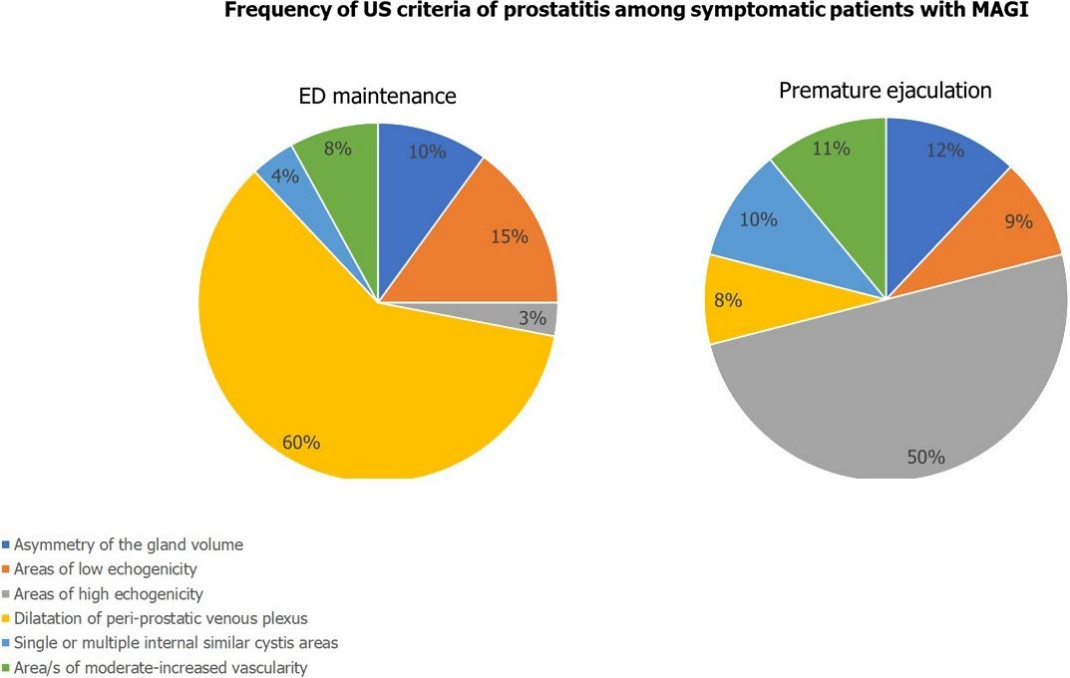
Frequency of US criteria of prostatitis among symptomatic patients with MAGI

**FIGURE 2:**
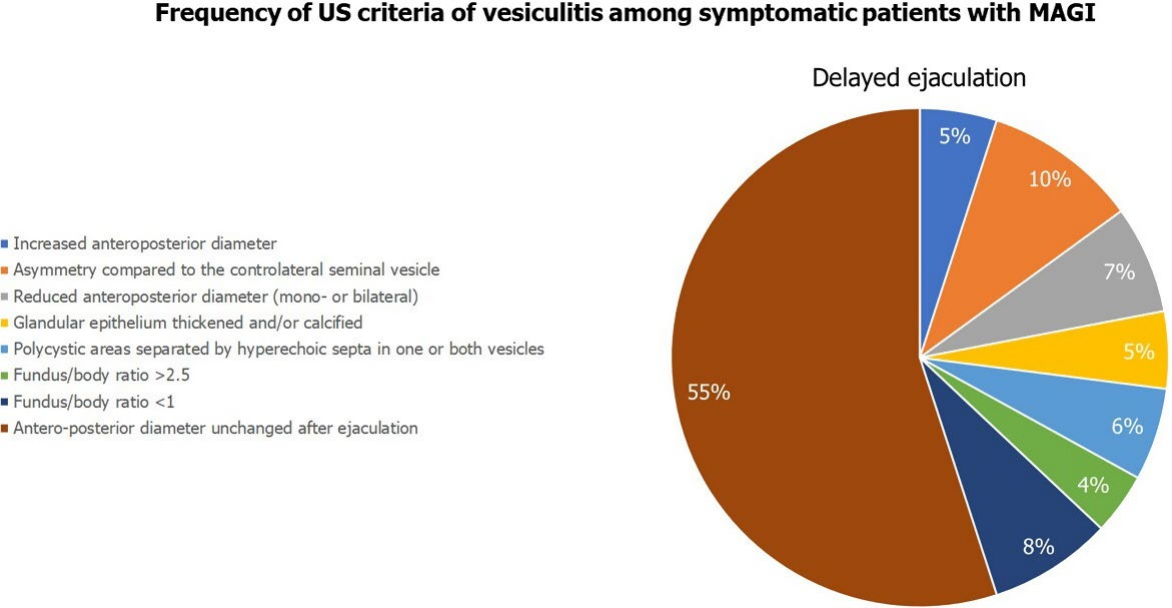
Frequency of US criteria of vesiculitis among symptomatic patients with MAGI

**FIGURE 3:**
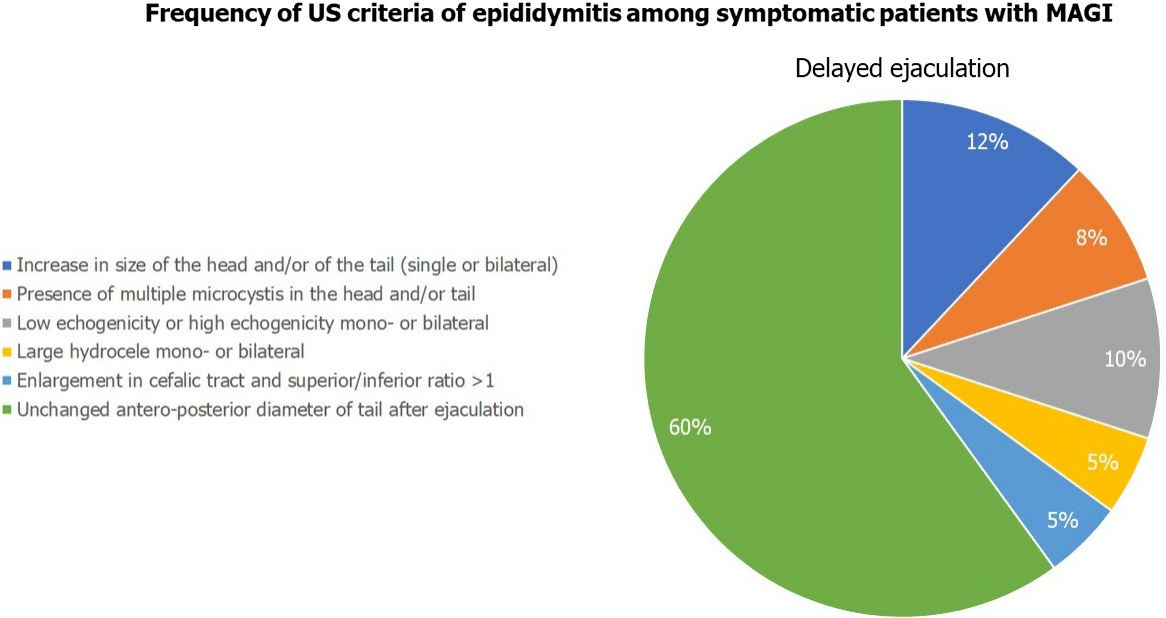
Frequency of US criteria of epididymitis among symptomatic patients with MAGI

Among the Group C patients, we restricted the analysis of the frequency of US criteria to patients who reported the following disorders to the questionnaire: erectile dysfunction in the achievement or maintenance phase, premature ejaculation, or delayed ejaculation. Figures 1, 2, 3 illustrate the results of this further analysis. Dilation of the periprostatic venous plexus was the most frequently detected US criterion in patients with erectile dysfunction in the maintenance phase (*p* < 0.05 compared to the other three groups). Areas of high prostatic echogenicity were the US criterion most frequently detected in patients with premature ejaculation (*p* < 0.05 compared to the other three groups), in 80% of cases (20/24 patients) detectable in the prostatic periurethral area. Finally, the absence of variation in the anteroposterior diameter of seminal vesicles and epididymal tail after ejaculation was the most frequently detected US criteria in patients with delayed ejaculation (*p* < 0.05 compared to the other three groups).

Finally, the green panels of Table 1 show the US criteria detected in patients with symptomatic MAGI with a significantly higher frequency than controls. Eleven US criteria out of twenty detectable were statistically more frequent in symptomatic MAGI compared to controls.

## 4 DISCUSSION

The results of this study suggest that the different array of symptoms of patients with MAGI associates with a specific US characterization. In addition, the presence of symptoms is linked to a significantly higher frequency of US MAGI criteria compared to patients without symptoms.

In particular, patients who report urinary disorders showed a greater frequency of areas of high echogenicity (almost always in the prostatic periurethral area) combined with areas of cystic‐like acinar ectasia. Patients who report spontaneous or post‐ejaculatory pain showed a higher frequency of polycyclic endoluminal areas in seminal vesicles, and, finally, patients with sexual dysfunction had a higher frequency of dilation of the periprostatic venous plexus.

Data analysis restricted to patients with MAGI who reported erectile dysfunction (in the achievement phase or in the maintenance phase) and/or ejaculatory disorders indicates that dilation of the periprostatic venous plexus was more frequent in patients with difficulty in maintaining an erection. The presence of areas of high echogenicity in the prostatic periurethral zone was more frequent in patients with premature ejaculation. Finally, patients with delayed ejaculation showed a high frequency of altered variation in the diameters of the seminal vesicles and epididymis after ejaculation.

US examination of the prostate, vesicular, and epididymal region in the clinical evaluation of patients with MAGI, while not contributing to the definition of diagnostic criteria (Factor A = anamnesis and physical examination; Factor B = characteristics of the prostatic fluid; Factor C = characteristics of the seminal fluid),^1^, ^2^ represents an important aid for the assessment of the anatomical extension of the inflammatory process, the bilateral involvement of the accessory sex glands, the identification of forms with better or worse clinical prognosis, and the characterization of patients belonging to specific clinical categories, such as diabetes, hypogonadism, papillomavirus infection.^15^, ^16^, ^17^, ^18^, ^19^

However, although more than 40 years have passed since the first diagnostic definition of MAGI^2^ and even though it is mentioned as one of the main causes of male infertility also by important international guidelines,^3^ this aspect, and in particular the application of US criteria, remains a subject of controversy. The US evaluation of the accessory sex glands still remains an operator‐dependent examination. In the case of the present study, all the US reports were performed by four different specialists trained to identify the MAGI US diagnostic criteria. Recently, the European Academy of Andrology published the results related to the standardization of US parameters of the epididymal region in healthy men.^20^ However, no multicenter study has been published so far on the US characteristics of patients with MAGI.

Not all andrology centers have an accurate selection of these patients, both due to an imprecise observance of the diagnostic criteria at the time of the first evaluation as well as for epidemiological issues.^1^ Very often, in fact, this category is confused with the mere presence of leukocytospermia or with urethritis or intercurrent cystitis.^13^ Concerning these aspects, it is worth considering that the chronic clinical course of MAGI could likely favor a different clinical interpretation, according to the phase of the first clinical evaluation.

In our opinion, the results of this study suggest important relapses in clinical practice for the management of symptomatic patients with MAGI. Men with urinary disorders are generally managed by urologists (after the age of 50), mainly for predominantly obstructive disorders secondary to prostatic hyperplasia.^21^ Fertile men affected by MAGI with US changes limited to the periurethral and transitional region of the prostate could simulate an obstructive urinary symptomatology and could benefit from pharmacological treatment with alpha blockers.^10^ The presence of areas of cystic‐like acinar ectasia in the prostate could contribute to persistent bacteriospermia, which suggest a role for an antibiotic and anti‐inflammatory therapeutic approach.^22^

Alpha blockers in urinary disorders associated with inflammatory prostatic disease represent an alternative option to antibiotics, anti‐inflammatories, topical steroids, and herbal medicine. The effectiveness of the treatment is highly controversial, and the profile of possible side effects (retro‐ejaculation; with the exception of alfuzosin, hypotension, dizziness) prevents the clinician from prescribing them in younger patients.^21^, ^23^ No studies have evaluated the efficacy of these drugs in patients with MAGI according to the US characterization. As an example, it is not clear whether treatment with alpha blockers may have a greater efficacy in patients with hyperechoic areas in periurethral prostatic region than patients without symptoms or US alterations in the peripheral region of the prostate.

Patients who report spontaneous or post‐ejaculatory pain in association with the US findings of seminal vesicles with polycyclic endoluminal areas may benefit from a decontracting and spasmolytic drug treatment aimed at resolving or reducing the images detected before the start of therapy. In clinical practice, these patients empirically diagnosed as chronic scrotal pain syndrome are often inappropriately treated with antibiotics without evidence of bacterial infection.^24^

The use of flavoxate in association with anti‐inflammatory and fibrinolytic treatment could represent a valid option for patients with extension of the inflammatory process to the seminal vesicles, with the aim of observing the US changes by transrectal ultrasound.^25^ Flavoxate (contraindicated in patients’ obstructive uropathies of the lower urinary tract) is an anticholinergic with antimuscarinic effects. Its muscle relaxant properties may be due to a direct action on the smooth muscle rather than by antagonizing muscarinic receptors. There are no other studies in the literature regarding the possible therapeutic application in MAGI.

Finally, among patients suffering from erectile dysfunction, it would be useful to differentiate those who have difficulty in achieving from those who have difficulty in maintaining an adequate erection (potential anamnestic criterion for venous erectile dysfunction).^26^ For the latter, the US evaluation of the prostate‐vesicular region and a possible treatment of ectasias of the periprostatic venous plexus would also be useful in an expanded diagnostic and therapeutic framework.

For these patients, it could be proposed an interventional evaluation for a possible embolization of the periprostatic venous plexus which represents an option with few evidence in the scientific literature. Rebonato and colleagues evaluated 18 patients with erectile dysfunction secondary to venous insufficiency. In these patients, venous continence was achieved with anterograde embolization of the periprostatic venous plexus, using a combination of N‐butyl cyanoacrylate and endovascular coils. In treated patients, mean Erectile Function Questionnaire score improved from 10.5 ± 5.2 to 20.6 ± 8.4 after the procedure (*p* = 0.0069). At 3‐month short‐term follow‐up, clinical success defined by an end‐diastolic velocity of <5 cm/s on color Doppler was noted in 81% (13 of 16 patients). Of these 13 patients, seven patients had continued erectile function at the end of follow‐up, and the other 6 patients reported progressive diminishment in the benefit over time.^27^

A recent review and meta‐analysis examined the results of the Endovascular Therapy for Vasculogenic Erectile Dysfunction on 212 patients with erectile dysfunction secondary to veno‐occlusive dysfunction (VOD). The VOD cohort were treated either percutaneously (60.4%; *n* = 128) or after surgical exposure of the deep dorsal vein (33.5%, *n* = 71), or it was unspecified (6.1%; *n* = 13). The most common embolic used was n‐butyl cyanoacrylate (51.9%; *n* = 109). Meta‐analysis found an overall clinical success rate of 59.8% in VOD patients.^28^

Finally, the results coming from the present study must be interpreted with caution due to some shortcomings. Firstly, although the clinicians performing the US evaluation received the same training and education, it cannot be excluded some bias derived from the operator‐dependent nature of the US technique. In addition, the retrospective study design limits the strength of the analysis though the considerably high number of included patients is a strength of the study. Furthermore, the SI‐MAGI questionnaire was used in the present analysis since its sections perfectly fit with the purpose of the study. However, this is a not widely recognized and adopted questionnaire,^29^ thus providing reasons for taking the present findings with care. On the other hand, this is the first study suggesting the existence of a correlation between the US features and the presence of symptoms in patients with MAGI.

In conclusion, the data of the present study suggest that symptoms may associate with US signs in patients with MAGI. Also, specific US signs may associate with specific symptoms. Further studies are needed to understand whether patients with specific US signs may in turn benefit from a personalized therapeutic choice. This knowledge would open the way toward new US‐aided personalized therapeutic approaches Figure S2.

## CONFLICT OF INTEREST

All authors declare no competing interests.

## AUTHOR’S CONTRIBUTIONS

S.L.V., A.E.C., A.A. involved in conceptualization; S.L.V. and A.C. contributed to writing—original draft preparation; A.E.C.; R.A.C.; A.A. involved in writing—review and editing; R.C. and M.C. involved in visualization; L.M.M.; R.C.; F.B., M.C. contributed to data curation; R.A.C., A.C., R.C. involved in supervision; A.E.C. and S.L.V. contributed to project administration; All authors have read and agreed to the published version of the manuscript.

## Supporting information

Figure S1

Figure S2
