# Supplementary figures and images for: Ultrasound aspects of symptomatic versus asymptomatic forms of male accessory gland inflammation
Source: Andrology. 2021 May 6;9(5):1422–8. doi: 10.1111/andr.13014 (PMC8596874; doi:10.1111/andr.13014)

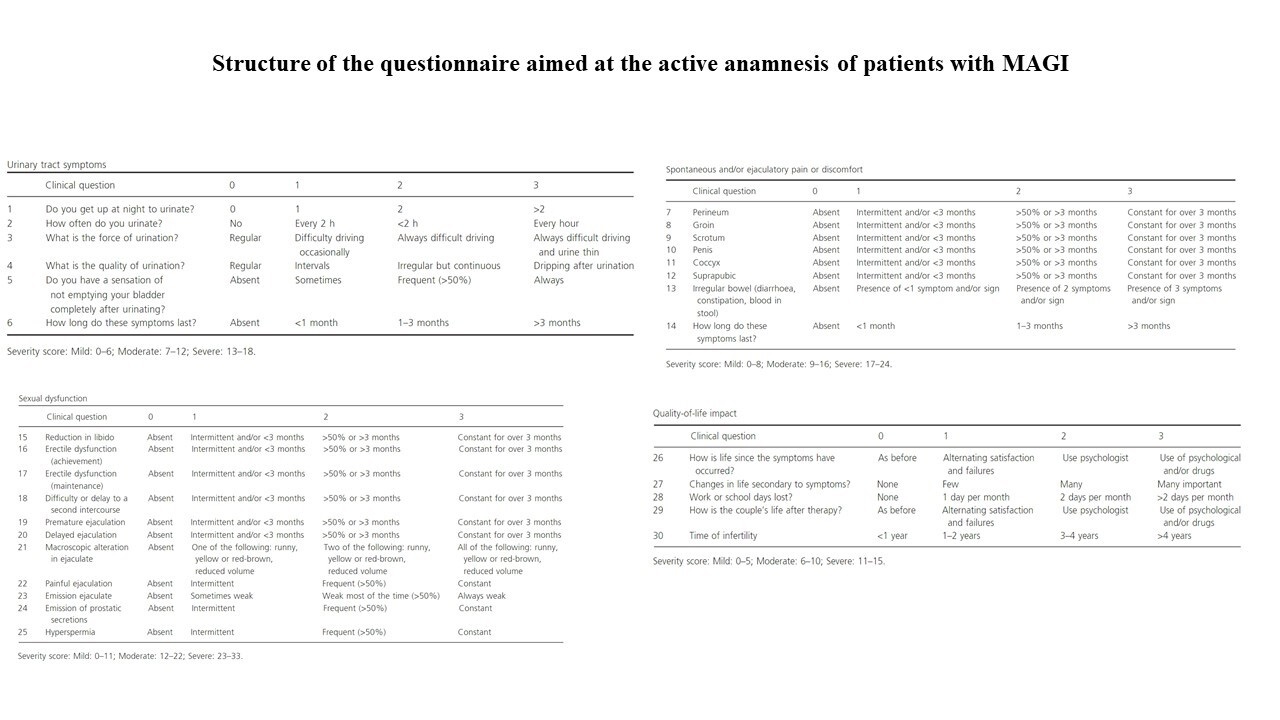

Supplement: Supplementary file 1 — Figure S1 [file ANDR-9-1422-s001.jpg]

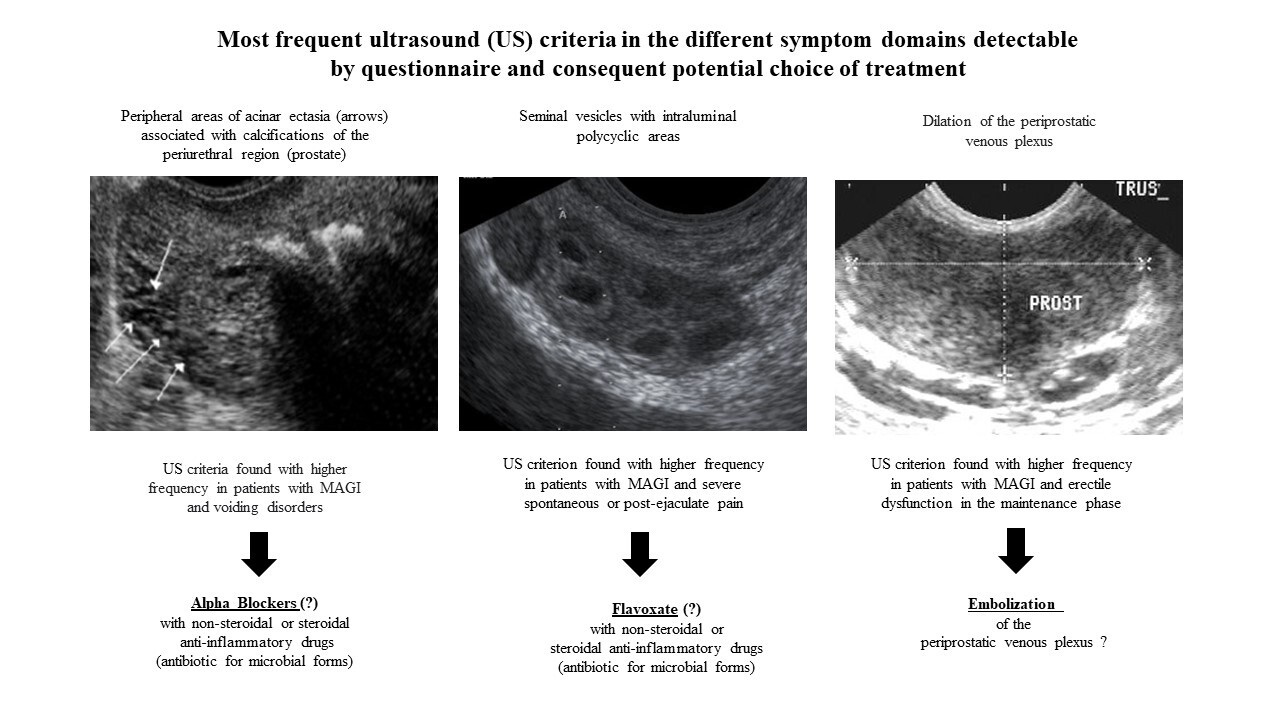

Supplement: Supplementary file 2 — Figure S2 [file ANDR-9-1422-s002.jpg]
